# Long-Term Results of a Phase I/II Clinical Trial of Autologous Mesenchymal Stem Cell Therapy for Femoral Head Osteonecrosis

**DOI:** 10.3390/jcm12062117

**Published:** 2023-03-08

**Authors:** Juan F. Blanco, Francisco J. Garcia-Garcia, Eva M. Villarón, Carmen da Casa, Helena Fidalgo, Miriam López-Parra, José A. Santos, Fermín Sánchez-Guijo

**Affiliations:** 1 Orthopaedic Surgery and Traumatology Department, University Hospital of Salamanca, 37007 Salamanca, Spain; 2 Biomedical Research Institute of Salamanca (IBSAL), 37007 Salamanca, Spain; 3 Regenerative Medicine and Cell Therapy Network Center of Castilla y Leon, Gerencia Regional de Salud, 47011 Valladolid, Spain; 4 Health Outcomes-Oriented Cooperative Research Networks in Advanced Therapies (RICORS TERAV), Instituto de Salud Carlos III, 28220 Madrid, Spain; 5 Department of Medicine, University of Salamanca, 37007 Salamanca, Spain; 6 Cell Therapy Unit, Hematology Department, University Hospital of Salamanca, 37007 Salamanca, Spain; 7 Radiology Department, University Hospital of Salamanca, 37007 Salamanca, Spain; 8 Department of Biomedical Sciences and Diagnostics, University of Salamanca, 37007 Salamanca, Spain

**Keywords:** osteonecrosis of the femoral head, core decompression, mesenchymal stromal cells, mesenchymal stem cells, MSC

## Abstract

(1) Background: Osteonecrosis of the femoral head (ONFH) is characterized by impaired vascularization with ischemia resulting in bone cell death, leading to the deterioration of the hip joint. Mesenchymal stem/stromal cells (MSCs) are an attractive potential therapeutic approach in this setting. The aim of this study is to evaluate the clinical improvement in terms of pain and quality of life, as well as the safety of the procedure during the follow-up of patients. (2) Methods: A Phase I–II Open-Label Non-Randomized Prospective clinical trial was conducted. Eight patients with idiopathic ONFH and stage < IIC in the ARCO classification were included. Four weeks before therapy, 40 mL of autologous bone marrow was obtained, and MSCs were expanded under Good-Manufacturing-Practice (GMP) standards. Study medication consisted of a suspension of autologous BM-derived MSCs (suspended in a solution of 5–10 mL of saline and 5% human albumin) in a single dose of 0.5–1 × 10^6^ cells/kg of the patient, administered intraosseously with a trocar and under radioscopic control. Per-protocol monitoring of patients included a postoperative period of 12 months, with a clinical and radiological assessment that included the visual analog scale (VAS), the Harris scale, the SF-36, and the radiological evolution of both hips. In addition, all patients were further followed up for eight years to assess the need for long-term total hip replacement (THR) surgery. (3) Results: Median age of patients included was 48.38 ± 7.38 years, and all patients were men. Autologous MSCs were expanded in all cases. There were no adverse effects related to cell administration. Regarding efficacy, both VAS and ODI scores improved after surgery. Radiologically, 12.5% of patients improved at the end of follow-up, whereas 50% improved clinically. No adverse effects related to the procedure were recorded, and none of the patients needed THR surgery within the first year after MSC therapy. (4) Conclusions: The use of autologous MSCs for patients with ONFH disease is feasible, safe in the long term, and potentially effective.

## 1. Introduction

Osteonecrosis of the femoral head (ONFH), also named avascular necrosis of the femoral head, is aseptic osteonecrosis of poorly clarified etiology. It is characterized by an alteration of the vascularization with ischemia that causes bone cell death, which may lead to hip joint deterioration by the development of a collapse and structural alteration. Various pathophysiological mechanisms have been postulated as leading to the development of bone necrosis, such as intravascular coagulation [1,2,3], lipid accumulation, and increased intraosseous pressure [1,3], as well as genetic factors [1,3,4] or anatomical alterations of the hip [1,5]. ONFH goes through various stages ranging from early stages, in which the structure of the femoral head is maintained and where it is possible to apply treatments that try to preserve the joint, to advanced stages, with the collapse of the femoral head and development of secondary osteoarthritis in which the treatment is usually a total hip replacement.

ONFH affects mostly young and active people between the 3rd and 5th decades of life, with a male: female ratio of approximately 4:1, and more than 50% of cases are bilateral [6]. Several diseases and a long list of risk factors have been associated with the development of ONFH, such as corticosteroid treatment, alcohol consumption, etc. [2,4,7,8,9]. Its prevalence has increased in recent years, partly due to the more widespread use of some drugs, such as corticosteroids, chemotherapy agents, or antiretroviral for HIV-AIDS [10], but also due to the increase in the prevalence of related diseases and other associated risk factors [1].

Early diagnosis of this disease is of utmost importance since the greatest effectiveness of treatments is obtained in the early stages. The main problem is that the clinical presentation is typically asymptomatic in these stages, and when the clinical presentation becomes more florid, the disease has usually advanced. Conventional radiographs show signs such as the crescent line, characteristic of the existence of subchondral fracture, as well as the loss of sphericity of the head due to collapse, joint narrowing, or arthrosis changes [11]. Magnetic resonance imaging (MRI) is the outreach diagnostic test [12]. MRI testing of ONFH shows the so-called double-lined sign, characterized by low-intensity signals at T1 and jointly low or high-intensity signals at T2, which reveals an interface between normal tissue and hypervascularized granulation tissue, distinctive of initial necrotic changes.

For the early phases of ONFH, asymptomatic and pre-collapse, there is still great controversy on the type of treatment to be followed. Non-surgical measures include physical therapy and pharmacological agents. They have the advantage that their use does not limit further treatments, and they can also be used as adjuvants. However, despite these notions, we should stress that, to date, there is no conservative treatment that unanimously demonstrates in studies a reversion of ONFH [13]. There is also no consensus on which surgical treatment should be performed depending on the stage of the disease, although total hip replacement is the most common procedure for the treatment of ONFH in the post-collapse phase, while core decompression is the most commonly used for the symptomatic pre-collapse phase. It was Fiat and Arlet [14,15] who, for the first time, focused on this type of treatment as a standard for the management of ONFH by reproducing their so-called “cervical-capital forage-biopsy”, later popularized by Hungerford as “Core-Decompression” (CD) [16]. However, the success of this procedure is highly dependent on the etiology of the condition and radiographic parameters such as lesion size, location, and the existence of collapse, and despite everything, the results in clinical practice have not been so satisfactory. Several authors advocate the use of bone marrow (BM) aspirate or concentrate in combination with forage in the treatment of femoral head osteonecrosis in its prolapse stages [17]. These have been somewhat frequently misnamed as stem cell therapy when the cellular product applied has not been shown to be mostly stem cells [18]. Mesenchymal stem (or stromal) cells (MSCs) are a well-defined cell type with the ability to differentiate into osteocytes and chondrocytes, as well as to induce an anti-inflammatory and immunomodulatory response, that may be of use in a number of clinical settings, including osteoarticular diseases [19]. In the current manuscript, we present the results of a phase I/II clinical trial on the use of autologous MSCs combined with cervical-capital forage for femoral osteonecrosis with a long-term follow-up (eight years).

## 2. Materials and Methods

### 2.1. Clinical Trial Characteristics

We conducted a prospective, open-label, non-randomized, phase I/II clinical trial of the treatment of idiopathic osteonecrosis of the femoral head by performing a forage together with the administration of autologous MSCs.

The whole study was conducted following the Declaration of Helsinki and was approved by the local Institutional Review Board (CEIm Area de Salud de Salamanca, reference code: 11/939) and the Spanish Medicines Agency (AEMPS; reference code: MUH/AEC) with EudraCT code 2011-005258-70. The trial was registered in www.clinicaltrials.gov with code NCT01700920 and sponsored by Fundación General de la Universidad de Salamanca.

The primary objective of the trial was to assess the safety and feasibility of the procedure. Secondary objectives were the assessment or the efficacy in terms of both clinical (visual analog scale (VAS), quality of life, Harris scale) and radiologic improvement (simple X-rays and magnetic resonance imaging-MRI).

### 2.2. Inclusion/Exclusion Criteria

The patients included in the study met the following inclusion criteria: age between 18 and 70 years, clinical and imaging (X-ray and MRI) diagnosis of idiopathic osteonecrosis of the femoral head and stage < IIC in the ARCO classification. Exclusion criteria were: patients who, in the investigator’s opinion, were not in an adequate situation to tolerate the procedure, clinical and anesthetic criteria contraindicating surgery (ASA IV-V), severe uncontrolled disease, pregnant women, patients with HIV+ infection, acute infection (in the previous 15 days) or chronic infection (other than HIV), having received previous treatment for osteonecrosis in the same hip, active or previous neoplastic disease (last five years), absence of informed consent or revocation of consent.

### 2.3. Study Procedures

Four weeks before treatment, each patient underwent a 40 mL BM aspiration from the posterior iliac crest, which was performed in the operating room under sterile conditions, sedation, and local anesthesia. BM cells were deposited in sterile bags with sodium heparin and sent to the Good-Manufacturing-Practice (GMP) Cell Production Unit to be processed immediately. Cells were produced according to the Investigational Medicinal Product Dossier (IMPD) with reference PEI-11-151, which was approved by AEMPS. In summary, BM mononuclear cells (BM-MNC) were obtained after density gradient centrifugation and further seeded into a tissue-culture flask (175 cm^2^) at an initial proportion of 3 × 10^7^ BM-MNC/flask in a medium with DMEM, 5% platelet lysate and expanded at 37 °C with 5% CO_2_. The medium was changed every 3–4 days, and cells were expanded until the therapeutic dose was obtained (maximum third passage). Quality controls and release criteria according to the IMPD included viability (>80%), flow-cytometric immunophenotyping (positive expression of CD44, CD90, and CD73, with negative expression of CD45, CD34, CD19, and CD14), multilineage differentiation ability (osteoblasts, adipocytes), karyotyping (normal) and mycoplasma detection (negative).

Study medication consisted of a suspension of autologous BM-derived MSCs (suspended in a solution of 5–10 mL of saline and 5% human albumin) in a single dose of 1 × 10^6^ cells/kg of the patient, administered intraosseously with a trocar and under radioscopic control.

The administration of the therapeutic product was based on a surgical procedure already described in the literature [17] and popularized by Fiat and Arlet. Thus, the patient was anesthetized and placed in supine decubitus on the surgical traction table. An incision of approximately 2 cm is made just at the base of the greater trochanter, dissecting until the external cortex of the femur is reached. A K-wire is introduced through this point, placing it at a maximum distance of 2–3 mm concerning the articular cartilage, and if the direction of the needle, after checking with fluoroscopic in AP and axial projections, was directed to the injured area of the femoral head, the external cortex is perforated using a cannulated drill. A trocar tip is introduced until the necrotic area is reached, and the cell suspension is administered through the trocar slowly over a few minutes. It is possible to inject the entire product since the necrotic zone has intertrabecular spaces that can be filled with the aspirate. Once the suspension injection is finished, the trephine or trocar needle is reintroduced to minimize the leakage of the cellular product through the trocar.

The follow-up of the patients established in the clinical trial protocol was one year and was carried out in four visits at one, three, six, and twelve months. In each visit, the following data were collected: clinical parameters of pain by serial evaluation of the visual analog pain scale (VAS), of function by evaluation of the Harris scale, and quality of life by evaluation of the SF-36 questionnaire the possible variation in work status, incapacity, change of activity or return to work was also evaluated.

For the Harris scale, the following parameters were considered: patients’ pain level, hip function, absence of deformity, and hip mobility. The SF-36 questionnaire was used to consider the physical function of the patients, their physical problems, their bodily pain, and their vitality/energy/fatigue, as well as their mental health, social role, and emotional problems, along with a general perception of their health status, and a global vision of physical and emotional health.

Regarding imaging assessment during follow-up, different X-ray evaluations of both hips, AP and axial, were performed at all visits, and MRI on the last two. Images were evaluated by a radiologist specializing in the musculoskeletal system, determining whether there had been an improvement, worsening, or stabilization of the ONFH according to standard radiological parameters. In addition, the MRI images were used to determine the stage according to the ARCO classification, calculating the relative volume of the necrotic lesion and expressing it as a percentage of the total femoral head and, using the pre-intervention test, comparing its evolution throughout the study. Therapeutic failure was defined by the presence of at least one of the following circumstances: worsening of painful symptomatology, progression of necrosis to collapse, or the need for total hip arthroplasty.

In addition to per-protocol established follow-up, patients were subsequently followed with routine visits for up to eight years to assess survival. The need for THR surgery or any other potentially related adverse events was also collected in their medical records.

### 2.4. Statistical Analysis

Statistical analysis was performed using the IBM^®^ SPSS^®^ Statistics program (v.26). Descriptive statistics included mean and standard deviation (SD). The normality of sample distribution was defined by the Shapiro–Wilk test, showing that all variables were distributed according to a Gaussian distribution. In order to compare patients over the follow-up time, the Student’s t-test for paired samples was used on quantitative variables, and Cochran’s Q test was used for paired categorical variables. In all cases, *p* ≤ 0.05 was considered statistically significant.

## 3. Results

### 3.1. Patient’s Characteristics

A total of eight male patients were enrolled in the study, and the median age at diagnosis was 48.38 ± 7.38 years (range 39–62 years). The mean body mass index (BMI) was 27.38 (only one subject had a normal BMI, and the remaining were overweight). Fifty percent had previous comorbidities, but none were related to osteonecrosis. Fifty percent had dyslipidemia, and (*n* = 3) 37.5% had hypertension.

Frequent alcohol and smoking consumption were declared in 7 (87.5%) and 6 patients (75%), respectively. Regarding the distribution of alcohol ingestion, 75% could be considered regular drinkers, with more than 300 mL of alcohol per week. Two patients (25%) were receiving corticosteroids at the time of inclusion. It was observed that at the time of diagnosis, all patients were under analgesic treatment, and only two patients (25%) maintained their daily work activity, while the rest were unemployed. At study entry, the percentage of osteonecrosis involvement in both hips was 75%. That is, it was present in six of the eight patients studied.

### 3.2. Analysis of Adverse Events and Feasibility of the Procedure

The primary objective of the clinical trial was to assess the safety and feasibility of the procedure during the entire post-infusion per-protocol follow-up of the patients (12 months). There was no reaction or complication related to the cell product or the surgery during the one-year follow-up, and the cell collection, ex-vivo expansion in GMP conditions and further intrabone administration of the cell therapy medicinal product were possible in all cases.

### 3.3. VAS and Harris Questionnaire’s’ Results

Concerning the results of the questionnaires, in the VAS, we observed how at the beginning, the minimum value of pain was already above half, with a minimum of 6 and a maximum of 10 (7.94 ± 1.50). Throughout the post-infusion follow-up, a decrease in this subjective sensation of pain was observed, reaching, in some cases, complete remission. According to these data, there seems to be an almost immediate improvement in the perception of pain after surgery (5.75 ± 1.17, *p* = 0.018), which slightly worsened in the third month (6.37 ± 2.37, *p* = 0.029) and decreased again after the sixth month (5.88 ± 2.84, *p* = 0.053) until the lowest values are observed at one year of follow-up (5.29 ± 3.55, *p* = 0.050). All different scores, and intra-group values, are shown in Table 1.

For the Harris questionnaire, in the “Pain” section, we observed an improvement throughout the study, with a significant difference of more than eight points in the mean between the baseline (16.25 ± 11.88) and final values (24.62 ± 15.37), *p* = 0.017. At baseline, we found mean values of 16.25 ± 11.87, while at the end of the follow-up, the value rose to 24.62 ± 15.37, *p* = 0.017. Regarding the “Function” section, we observed an improvement in the general functional capacity of the patients who underwent treatment, although the *p*-value is not statistically significant. An objectively measurable recovery of approximately five points was observed, with an initial value of 17.62 ± 9.00, reaching 22.25 ± 10.19 at the end of the follow-up, *p* = 0.097. In the “Activity”, it was observed that during the 12-month follow-up, the trend towards an increase in these activities was maintained, going from a mean value of 8.25 ± 2.31 at the beginning to 9.12 ± 4.02 at the end, *p* = 0.500.

In the category of the absence of deformity, there was no variation throughout the study since no patient met the criteria to be considered a fixed deformity in flexion, adduction, internal rotation/flexion, or dysmetria greater than 3.2 cm. In terms of mobility, it should be noted that despite presenting considerable joint mobility from the initial situation (5.63 ± 0.74), from the 6th month of treatment, the values increased to the maximum for all patients (6.00 ± 0.00), *p* = 0.197. At the 12-month follow-up, the mean value remained stable.

Finally, in total terms of the Harris questionnaire, there was a significant improvement in the mean values from the baseline, going from a mean of 52.00 ± 18.02 to 67.00 ± 27.55, despite obtaining one of the lowest values in one patient at one year (*p* = 0.014). Concerning the SF-36 questionnaire, the baseline values for physical status were well below the population mean and somewhat better for mental status, although also below the mean. There was a continuous increase in both the overall physical and mental health values throughout the follow-up.

### 3.4. Radiological Findings

**Figure 1:**
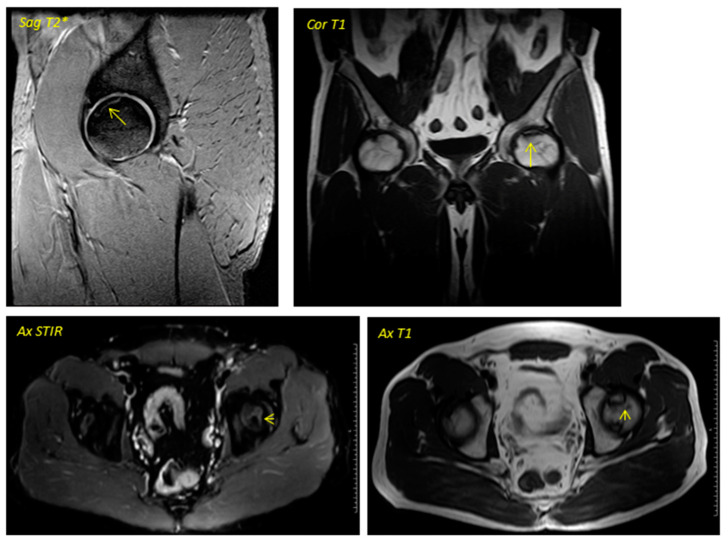
Pre-surgical image corresponding to a 38-year-old male performed in December 2013. The geographic area in the superior anterior region of the left femoral head is delimited by a hypointense line in T1 sequences and hyperintense in T2 sequences in relation to avascular necrosis. No loss of sphericity of the femoral head or joint effusion is evident. It corresponds to type II in the Association Research Circulation Osseous (ARCO) classification.

**Figure 2:**
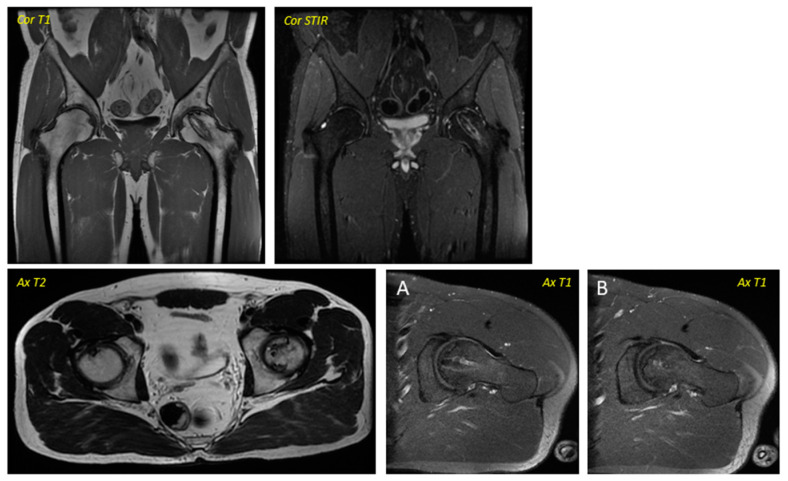
Post-surgical Magnetic Resonance Imaging corresponding to the patient in Figure 1 was performed in April 2016. Ax T1 (axial T1 plus contrast plus fat suppression–(A) Axial plus caudal incidence and axial (B) plus cranial incidence). Trocar entry is visualized in all images.

Regarding the imaging studies, several findings were observed in the radiographic evolution during the 12-month follow-up (Figure 1 and Figure 2). First, changes in the direction appeared in all cases from the sixth month, not being evident in earlier stages. Second, radiographic stabilization was achieved in 62.5% of the patients (*n* = 5) during the first year after surgery. Third, two patients (25%) showed the progression of the necrotic zone, while in one (12.5%), signs of re-ossification were evident. Despite observing descriptive differences, the *p*-value was not statistically significant for the latter parameter (*p* = 0.317).

Regarding the dynamics in the MR images, two patients were baseline in stage IA of the ARCO classification (25%), two in IIA (25%), and four in IIB (50%). In the same way, a stabilization in the staging of the post-surgery images was observed in seven of the eight patients studied (87.5%), with only one case (12.5%) evolving from grade IIB to grade IIIA, presenting subchondral collapse of the femoral head, which was already evident from the sixth month onwards. Descriptively, changes were observed, but they were not statistically significant. No significant variability was observed in the imaging findings between patients.

### 3.5. Clinical Parameters

The clinical improvement found in these patients throughout the post-infusion follow-up was striking, especially in terms of functionality and reduction of pain and discomfort. Already from the first month after surgery, there was an improvement in the symptoms in all the subjects except one, who remained stable. In the third month, 100% reported a substantial improvement. Regarding the sixth month, and as indicated in the evolution of the rest of the parameters, it seems to be the point of inflection to a positive or negative outcome. In this case, a favorable evolution predominates in the patients. Thus, five (65.5%) presented clinical improvement, two (25%) found no change, and only one patient (12.5%) worsened since the previous visit. At one year and the end of follow-up, four patients (50%) further increased the sensation of symptomatic relief, two (25%) stabilized concerning the previous visit, and another two (remaining 25%) did not improve after cell therapy. Overall, and analyzing the general condition perceived by the patient during the consultations, a clinical improvement with the treatment was observed in 75% of the patients.

Another important parameter in the clinical evaluation was the mobility of the affected hip and its possible mechanical limitations due to osteonecrosis. An average improvement in the degrees measured in each of the movements was evident throughout the 12 months of follow-up. When comparing baseline flexion (116.87° ± 11.93°) with that at one month (122.50° ± 13.09°) and six months (127.50° ± 13.88°), there were statistically significant differences (*p* = 0.002, *p* =0.031, respectively). It was in these first months that an increase in hip flexion angle was observed. For hip abduction (26.25° ± 5.18°-baseline grades), an increase in angle was observed at one month (28.75° ± 5.84°, *p* = 0.033), six months (33.13° ± 5.30° *p* = 0.004), and one-year follow-up (35.00° ± 4.63°, *p* = 0.002). In contrast, for hip adduction (28.13° ± 4.58°-baseline grades), differences were statistically significant only at one-year follow-up (33.75° ± 5.82°, *p* = 0.038). Regarding the degrees of hip rotation, both external and internal, an increase in both angles was observed, but only for external rotation. A statistically significant increase was confirmed at six months (*p* = 0.026) and at one-year follow-up (*p* = 0.018). Overall, an increase comparing the baseline situation (221.25° ± 25.04°) and one-month follow-up (232.88° ± 24.91°, *p* = 0.032), six months (253.75° ± 32.38°, *p* = 0.011), and one-year follow-up (261.88° ± 32.83°, *p* = 0.004) after drilling surgery and infiltration of MSCs was observed.

### 3.6. Need for Surgery

Knowing that, after the initial diagnosis of ONFH, progression is rapid and the evolution to hip arthroplasty in many cases is imminent, another important objective of the study was to determine what percentage of the patients exposed to treatment ended up requiring indication for prosthetic surgery during the year of follow-up. As will be indicated in the next section, none of the included patients underwent THR surgery during the 12 months of per-protocol established follow-up.

### 3.7. Long-Term Follow-up

After an extended long-term follow-up of eight years, four out of the eight treated patients (50%) needed a THR surgery to replace the MSC-treated femoral head (Table 2) after a median of 574 days (range: 558–1857 days).

## 4. Discussion

The main contribution of the present work is not only to present the results of a trial in the initial phases of treatment with autologous MSCs in femoral osteonecrosis, an osteoarticular disease with a medical treatment that can be improved at present but also to provide long-term follow-up data (eight years), which is not common in any of the trials in this field.

Idiopathic ONFH is a progressive disease due to different pathophysiological mechanisms that are not well understood, such as extra or intravascular fatty plugging, micro-embolism, or intravascular coagulation, leading to a decrease or interruption of blood flow, which in advanced stages ends up destroying the hip joint if left untreated. In the early stages treatment aims to halt, or otherwise slow down, the progression towards a bony collapse of the femoral head, while in advanced stages CTA is the only option available with proven results. For all these reasons, it is striking how a disease that can account for around 10% of the indications for a total hip prosthesis, and in which there seems to be a slight increase in incidence in recent years, we have not been able to find a sufficiently effective treatment to halt its progression even in those cases in which it is diagnosed, despite the difficulty, in early stages. Under this premise, added to the inconsistent results that we have observed in our clinical practice with CD alone, supported by studies published by different authors [20,21], which show that the elimination and decompression of the necrotic zone are not sufficient from the pathophysiological point of view to stabilize or regenerate this condition, the idea of this study arises: to combine the classic treatment of CD with the new advances that are taking place in the field of cell therapy with MSCs.

Autologous MSCs were obtained from the bone iliac crest approximately four weeks before cell administration. Isolation and expansion were performed following an AEMPS-approved procedure to ensure that each patient received a final dose of 0.5–1.0 × 10^6^ cells/kg body weight (total dose was around 80 × 10^6^ MSCs for most patients). In this way, we obtain bonafide MSCs free of contamination with pro-inflammatory cells or with other cell types present in BM or in lipoaspirates, which is an advantage of our method and differentiates us from other studies where the sample is collected without an isolation or culture process, or where the number of implanted cells remains undetermined [22,23]. Pioneering studies in this field are those carried out by authors such as Hernigou [24,25], which began with the introduction of BM aspirate autograft through femoral tunneling in 1997, thus being the first report of the use of cell therapy as a treatment and later followed by authors such as Gangji [25]. In this sense, it is necessary to bear in mind that BM aspirates are not a cell therapy as such since we do not know the cell types they contain or their exact quantity. The same is true if platelet-rich plasma (PRP) is used. Therefore, although these works are in some way precursors, they cannot be considered cell therapy.

Regarding the source of MSCs, BM remains the most widely used. Even so, other sources are increasingly used and explored, such as adipose tissue, Wharton’s jelly from the umbilical cord, or placenta, among others [22,26]. In our study, we opted for BM-derived MSC based on their extensive use in osteoarticular diseases and specifically due to the prior use of BM aspirates in osteonecrosis, as just mentioned, together with our experience with this advanced therapy medical products in prior clinical trials.

The primary objective of the current prospective phase I-II clinical trial was to assess the safety and feasibility of the procedure. It must be stressed here that in all cases, BM-derived autologous MSCs were able to be obtained from iliac crest aspiration to the final MSC batches to be administered intrabone in each patient. The safety of autologous and allogeneic MSCs has been demonstrated throughout the published results on more than 3000 treated patients [27,28].

The potential risk of alloimmune reaction is reduced in allogeneic MSC due to the absence of expression of major histocompatibility complex class II molecules under basal conditions and their immunomodulatory capacity [23], and in the case of autologous cells is obviously not a concern. The MSC products used in our trial were tested for genetic and chromosomal alterations and microbial contamination before release, without any discarded product from any batch. Likewise, there were no complications of any kind during cell extraction, CD surgery, and infusion or postoperative follow-up related to the procedure. There was also no reaction or adverse effect on the cell product. This safety profile is in agreement with a prior report where autologous MSCs were administered with autologous bone grafts [29].

For the assessment of our clinical results, we based ourselves mainly on three global factors: the patient’s symptomatology and its evolution in clinical scores throughout the follow-up, the resulting changes in the imaging techniques, and, in the long term, the need to perform a THR surgery. Regarding the clinical situation, it should be emphasized that all but one of the subjects improved their pain in the first month post-procedure, and around the third month, all the patients reported a subjective improvement. This fact may be potentially related to the decrease in intraosseous hypertension that occurs after perforation of the lesion and consequent reduction of edema, as well as by the anti-inflammatory effect induced by MSCs themselves. At one year and the end of the per-protocol follow-up, half of the patients reported being clinically better than before surgery and satisfied with the outcome of the procedure, two patients (25%) seemed to be stabilized, similar to their previous state, and two (25%) were worse despite the initial symptomatic relief. Thus, these results are comparable to those found in the studies of Gangji or Hernigou [25,30]. Overall, there was again in the total degree of mobility of the affected hips. The study of the questionnaires also yielded concordant results with the clinical data, the VAS, the Harris questionnaire, and the health and quality of life assessment scale (the SF-36) in global terms improved. In most studies reported to date, the improvement in clinical symptomatology and functionality of the hip is a constant, although the values are notably higher than those obtained in our clinical trial. Sen et al. [31], for example, found results over 76 points on the HHS at 12 months, and Wu et al. [32] found a mean of 84.66 in the HHS and a VAS of 1.91 after nine months of treatment.

As for the imaging tests, in the radiographic and MRI series, it is not until the sixth month after MSC therapy that radiological changes are noticeable, remaining stable until the end of the twelve-month follow-up. Other studies have also shown improvement and stabilization of the images from MRI, although we observed more favorable values [33]. In this regard, in the work of Tabatabaee et al. [34], where of the 14 patients treated with CD and MSCs, two patients progressed from stage III to stage II, and one patient from stage II to stage I of ARCO after 24 months of follow-up, the rest remaining in the same stage. Kang et al. [35], with a follow-up period of approximately four years, estimate 38% (19 hips) progression in ARCO grades despite receiving parameters such as lesion size, location, degree of the classification, time to diagnosis, and certain etiological factors that are not completely known. But it seems that the size of the necrotic lesion is the most important predictive factor. Hernigou [25] estimates a collapse of 80% of femoral heads with necrotic involvement of between 10–20% and 50% in those with less than 10% necrotic involvement [36]. In our study, 50% had less than 15% involvement, and the other 50% had between 15% and 30%, one of which was the one that progressed to collapse in the trial.

Finally, regarding the need for THR surgery in our study, although results have to be taken with caution due to the small sample size of a phase I-II study, none of the patients needed a hip prosthesis during the first year of per-protocol follow-up, and with our extended long term results to specifically assess this item, the median time for THR surgery (for the 50% that eventually needed on the treated femoral head) was 576 days, which is substantially better than the expected outcome for this type of advanced disease patients. Nevertheless, since our trial was a phase I/II, the clinical relevance of higher doses of MSCs should be explored in future phase III randomized trials. The available data on the need for total hip replacement after biopsy-forage in femoral head osteonecrosis is variable and present difficult interpretation due to the different methodologies used, femoral head osteonecrosis stage and the cell source used. In this sense, the present work uses a known and well-characterized source. Tomaru et al. [37] report a reconversion rate of 9.6% at 33 months using a bone marrow concentrate. In a comparative study between performing forage biopsy alone or combined with the application of a cellular product (BM concentrate), Kang et al. [35] found a conversion rate to THR of 50% in cases of isolated forage versus 20% when combined with the cellular substrate, in both cases with a 4-year follow-up. The conversion rate in total hip prostheses seems to be lower when decompression is combined with the application of a cellular product [38]. Despite the variability of the published data (but based on Gangji et al. results [39]), and in an attempt to estimate the number of patients needed to observe significant differences in the need for total hip replacement after 3-years of follow-up in phase III randomized trial comparing patients treated with forage plus MSCs compared to forage alone, with a power of 80% and a patient loss of 3%, each arm would have to include approximately 60 patients. As a final additional comment, the importance of the technical aspects of this therapeutic strategy should be noted: as Gómez-Barrena et al. have reported in the results of the REBORNE trial with comparable results to those of our trial, that regarding the forage drilling technique, it is recommended to place it in the weight-bearing area in the coronal plane and avoid the varus position [40].

The main limitations of our study were found when assessing and analyzing the effect of MSCs in such a short series of patients, given the characteristics of this phase I/II clinical trial. Likewise, it is known that the efficacy of the process may be closely linked to the number of cells implanted, but the number of cells needed to stabilize or improve a necrotic area is unknown [25,39]. This is one of the major limitations when analyzing the literature. There is great variability concerning the cell therapy used, the cell type and the way it is obtained, isolated and expanded, its qualitative and quantitative management, and the method of implantation. Thus, drawing comparative conclusions between the various studies for the evaluation of results and the relative efficacy between different cell therapy strategies should be performed with caution. Together with the low number of patients, the lack of long-term imaging results (eight years of radiographs or MRI) is also an important limitation. All these aspects should be assessed in the phase III randomized trial suggested previously, which would really confirm the real efficacy of this combined treatment strategy of forage and MSCs versus the current forage-only standard treatment.

## 5. Conclusions

The results of the current phase I-II clinical trial show that the administration of autologous BM-derived MSCs for femoral head osteonecrosis is feasible and safe. There are improvements in clinical and radiological parameters, as well as a potential delay in the need for total hip replacement, but they cannot be attributable alone to cellular treatment. Therefore, these preliminary results should be confirmed in phase III clinical trials and compared to forage alone as a control arm.

## Figures and Tables

**Table 1:** Patient scores throughout the follow-up period. Numerical variables are indicated by mean ± sd and categorical variables by percentages. The intra-group value is shown, referring to the baseline value (and, in some cases, the first month of follow-up).

SCORE	Baseline		1-Month	3-Months	6-Months	12-Months
VAS (numerical)	8.13 ± 1.49		5.75 ± 1.16	6.37 ± 2.37	5.87 ± 2.83	5.06 ± 3.34
p (intra-group) respect to baseline			0.018 *	0.029 *	0.053	0.051
HARRIS (pain)	16.25 ± 11.87		22.50 ± 8.86	25.50 ± 14.80	21.25 ± 14.57	24.62 ± 15.37
p (intra-group) respect to baseline			0.049 *	0.036 *	0.033 *	0.017 *
HARRIS (function)	17.62 ± 9.00		18.75 ± 11.27	21.00 ± 8.83	22.25 ± 6.31	22.25 ± 10.19
p (intra-group) respect to baseline			0.786	0.322	0.066	0.158
HARRIS (activity)	8.25 ± 2.31		8.75 ± 2.18	8.25 ± 3.05	8.50 ± 2.97	9.12 ± 4.01
p (intra-group) respect to baseline			0.598	0.999	0.756	0.500
HARRIS (lack of deformity)	4.00 ± 0.00		4.00 ± 0.00	4.00 ± 0.00	4.00 ± 0.00	4.00 ± 0.00
p (intra-group) respect to baseline			0.999	0.999	0.999	0.999
HARRIS (mobility)	5.62 ± 0.74		5.87 ± 0.35	5.62 ± 0.74	6.00 ± 0.00	6.00 ± 0.00
p (intra-group) respect to baseline			0.451	0.999	0.197	0.197
HARRIS (total)	52.00 ± 18.02		60.00 ± 18.74	64.37 ± 23.00	62.00 ± 21.84	67.00 ± 27.54
p (intra-group) respect to baseline			0.188	0.062	0.023 *	0.014 *
SF36/Physical Function	38.75 ± 22.95			36.25 ± 19.04	43.75 ± 27.61	52.50 ± 33.59
p (intra-group) respect to baseline				0.743	0.516	0.097
SF36/Physical Problems	0.00 ± 0.00			6.25 ± 11.57	18.75 ± 34.71	43.75 ± 49.55
p (intra-group) respect to baseline				0.170	0.170	0.041 *
SF36/Mental Health	50.00 ± 29.62			58.00 ± 30.68	61.50 ± 21.26	69.00 ± 30.37
p (intra-group) respect to baseline				0.054	0.091	0.013 *
SF36/Emotional Problems	62.5 ± 51.75			57.14 ± 53.45	71.43 ± 48.80	60.00 ± 54.77
p (intra-group) respect to baseline				0.129	0.356	0.147
SF36/Vitality, Energy or Fatigue	35.62 ± 28.71			481.2 ± 24.33	50.00 ± 23.29	57.50 ± 31.51
p (intra-group) respect to baseline				0.011 *	0.020 *	0.007 *
SF36/General Perception Health	38.75 ± 13.02			42.5 ± 13.09	42.5 ± 11.64	57.5 ± 31.50
p (intra-group) respect to baseline				0.413	0.320	0.584
SF36/Physical Health Global	103.75 ± 55.28			82.50 ± 45.96	110.00 ± 58.95	225.00 ± 161.17
p (intra-group) respect to baseline				0.421	0.893	0.153
SF36/Mental Health Global	181.29 ± 135.22			261.00 ± 143.88	254.75 ± 155.01	286.00 ± 170.99
p (intra-group) respect to baseline				0.546	0.724	0.408
SF36/Total	414.00 ± 161.95			371.75 ± 89.38	279.00 ± 183.50	399.50 ± 346.59
p (intra-group) respect to baseline				0.188	0.062	0.014 *
Hip Flexion	116.88 ± 11.93		122.50 ± 13.09	116.88 ± 29.63	127.50 ± 13.89	124.38 ± 16.35
p (intra-group) respect to baseline			0.026 *	0.999	0.031 *	0.111
Hip Abduction	26.25 ± 5.18		28.75 ± 5.84	28.75 ± 5.84	33.13 ± 5.30	35.00 ± 4.63
p (intra-group) respect to baseline			0.033 *	0.451	0.004 *	0.002 *
Hip Adduction	28.13 ± 4.58		28.13 ± 5.94	29.38 ± 4.17	33.13 ± 6.51	33.75 ± 5.82
p (intra-group) respect to baseline			0.999	0.598	0.068	0.038*
Hip External Rotation	25.00 ± 8.0.2		27.25 ± 6.02	26.88 ± 6.51	32.50 ± 4.63	34.38 ± 4.17
p (intra-group) respect to baseline			0.125	0.528	0.026 *	0.018 *
Hip Internal Rotation	25.00 ± 8.45		26.25 ± 6.41	26.25 ± 7.44	27.50 ± 8.02	33.13 ± 6.51
p (intra-group) respect to baseline			0.351	0.775	0.470	0.061
Total Degrees of Mobility	221.25 ± 25.04		232.88 ± 24.91	226.88 ± 41.14	253.75 ± 32.38	261.88 ± 32.83
p (intra-group) respect to baseline			0.032 *	0.766	0.011 *	0.004 *
Radiography	Improvement				12.5%	12.5%
	Stabilization		100%	100%	62.5%	62.5%
	Worsening				25%	25%
p (intra-group) respect to the first month				0.999	0.317	0.317
Magnetic Resonance Imaging (ARCO)	IA	25%			25%	25%
	IIA	25%			25%	25%
	IIB	50%			37.5%	37.5%
	IIIA				12.5%	12.5%
p (intra-group) respect to baseline					0.467	0.467
Clinic	Best		87.5%	100%	62.5%	50%
	Same		12.5%		25%	37.5%
	Worst				12.5%	12.5%
p (intra-group) respect to the first month				0.317	0.157	0.083

Visual Analog Scale (VAS); Harris Hip Score; Short Form 36 Health Survey Questionnaire (SF-36); Association Research Circulation Osseous (ARCO); Statistically significant *p*-values are marked by *.

**Table 2:** Shows the date the Autologous Mesenchymal Stem Cells (MSC) were administered, the side of the hip on which they were injected, the time to Total Hip Replacement (THR), and the hip side replaced.

ID	Date	Side	Time to THR (Days)	Side to THR
1	10 August 2012	Right	558	Right
2	20 February 2013	Right		
3	10 April 2013	Right	576	Right
			790	Left
4	9 May 2013	Left	1857	Left
5	12 August 2013	Left		
6	23 September 2013	Left	574	Left
			1346	Right
7	2 June 2014	Left	574	Right
8	8 October 2014	Left		

## Data Availability

The data supporting this study are available from the corresponding author upon reasonable request.
